# Diffusion-weighted MRI to determine response and long-term clinical outcomes in muscle-invasive bladder cancer following neoadjuvant chemotherapy

**DOI:** 10.3389/fonc.2022.961393

**Published:** 2022-11-14

**Authors:** Shaista Hafeez, Mu Koh, Kelly Jones, Amir El Ghzal, James D’Arcy, Pardeep Kumar, Vincent Khoo, Susan Lalondrelle, Fiona McDonald, Alan Thompson, Erica Scurr, Aslam Sohaib, Robert Anthony Huddart

**Affiliations:** ^1^ Division of Radiotherapy and Imaging, The Institute of Cancer Research, London, United Kingdom; ^2^ Urology Unit, The Royal Marsden National Health Service (NHS) Foundation Trust, London, United Kingdom; ^3^ Department of Radiotherapy, The Royal Marsden NHS Foundation Trust, London, United Kingdom; ^4^ Department of Diagnostic Radiology, The Royal Marsden National Health Service (NHS) Foundation Trust, London, United Kingdom

**Keywords:** muscle invasive bladder cancer (MIBC), neoadjuavant chemotherapy, MRI, diffusion weighted magnetic resonance imaging (DWI), imaging biomakers

## Abstract

**Objective:**

This study aims to determine local treatment response and long-term survival outcomes in patients with localised muscle-invasive bladder cancer (MIBC) patients receiving neoadjuvant chemotherapy (NAC) using diffusion-weighted MRI (DWI) and apparent diffusion coefficient (ADC) analysis.

**Methods:**

Patients with T2-T4aN0-3M0 bladder cancer suitable for NAC were recruited prospectively. DWI was performed prior to NAC and was repeated following NAC completion. Conventional response assessment was performed with cystoscopy and tumour site biopsy. Response was dichotomised into response (<T2) or poor response (≥T2). Patients proceeded to either radical cystectomy or chemo-radiotherapy as standard of care. Tumour ADC values were calculated for all b-values (ADC_all_) and high b-values (ADC_b100_). Mean ADC, percentiles, skew, kurtosis, and their change (ΔADC and %ΔADC) were determined. Threshold predictive of response with highest specificity was ascertained using receiver operating characteristic (ROC) analysis. Median overall survival (OS), bladder-cancer-specific survival (bCSS), progression-free survival (PFS), and time to cystectomy were estimated using Kaplan–Meier method. Significant area under the curve (AUC) cut points were used to determine relationship with long-term endpoints and were compared using log-rank test.

**Results:**

Forty-eight patients (96 DWI) were evaluated. NAC response was associated with significant increase in mean ΔADC and %ΔADC compared to poor response (ΔADC_all_ 0.32×10^−3^ versus 0.11×10^−3^ mm^2^/s; p=0.009, and %ΔADC_all_ 21.70% versus 8.23%; p=0.013). Highest specificity predicting response was seen at 75th percentile ADC (AUC, 0.8; p=0.01). Sensitivity, specificity, positive predictive power, and negative predictive power of %ΔADC_b100_ 75th percentile was 73.7%, 90.0%, 96.6%, and 52.9%, respectively. %ΔADC_b100_ 75th percentile >15.5% was associated with significant improvement in OS (HR, 0.40; 95% CI, 0.19–0.86; p=0.0179), bCSS (HR, 0.26; 95% CI, 0.08–0.82; p=0.0214), PFS (HR, 0.16; 95% CI, 0.05–0.48; p=0.0012), and time to cystectomy (HR, 0.19; 95% CI, 0.07–0.47; p=0.0004).

**Conclusions:**

Quantitative ADC analysis can successfully identify NAC response and improved long-term clinical outcomes. Multi-centre validation to assess reproducibility and repeatability is required before testing within clinical trials to inform MIBC treatment decision making.

**Advances in knowledge:**

We successfully demonstrated that measured change in DWI can successfully identify NAC response and improved long-term survival outcomes.

## Introduction

Curative treatment options for patients with localised muscle-invasive bladder cancer (MIBC) includes either radical cystectomy or radiotherapy delivered with systemic radiosensitisation (1–4). Neo-adjuvant cisplatin-based combination chemotherapy is offered to patients prior to radical treatment given improved overall survival benefit (5, 6). Accurate prediction of neo-adjuvant chemotherapy (NAC) response is required to avoid delays in the effective treatment of potential non-responding tumours. Consensus molecular classification of MIBC has identified six subtypes, which differ in oncogenic mechanism, histological characteristics, and clinical outcomes (7). Despite this, no molecular biomarkers have yet been identified, successfully validated, and prospectively tested to predict NAC response such that they could be routinely applied clinically to inform patient stratification (8).

Imaging biomarkers are used in cancer detection, characterisation, and response assessment (9). In bladder cancer, multi-parametric MRI (mpMRI) has been evaluated for local staging in three meta-analyses (10–12). Performance of mpMRI exceeds both CT imaging and trans-urethral resection of the bladder tumour (TURBT) (13–16). A qualitative mpMRI scoring system, the Vesical Imaging-Reporting and Data System (VI-RADS) for newly diagnosed bladder tumours as seen on T2-weighted sequences, diffusion-weighted imaging (DWI), and dynamic contrast enhancement (DCE) images has been developed (17). However, quantitative analysis may provide additional insights to bladder tumour characteristics (18, 19).

DWI contrast is dependent on the inhibitory effect of cell membranes to the random motion of water molecules. The higher cellular density of tumours restricts water molecule diffusion compared to normal tissues. These diffusion characteristics can be captured quantitatively by several metrics; most commonly the mean apparent diffusion coefficient value (ADC) is used. ADC measurements have association with pathological features that can distinguish between bladder T stage, tumour grade, size, and biological proliferation markers (19–23).

Change in ADC has been used to assess treatment response in other tumours (24–28). Following successful treatment, a decrease in cellularity at the tumour site results in ADC increase. Conventional local bladder treatment response assessment is with cystoscopy. DWI, therefore, has potential for non-invasive local bladder treatment response assessment (23, 29). In this study, we evaluated the role of quantitative DWI analysis to assess local treatment response and long-term survival outcomes for MIBC patients receiving NAC.

## Materials and methods

### Study population

This is a pooled exploratory analysis of patients recruited prospectively to single-centre clinical research and ethics-committee-approved protocol conducted in accordance with Good Clinical Practice guidelines.

All patients had initial TURBT and pathological confirmation of MIBC, and were staged with CT scan of the chest, abdomen, and pelvis. Those with T2-T4aN0-3M0 disease according to the American Joint Committee on Cancer (7th edition) of any histological subtype scheduled to receive NAC were considered eligible if they had no contradiction to MRI or objection to follow-up that included repeat MRI and cystoscopy with biopsy.

### Treatment and conventional response assessment

Suitable patients received platinum combined with NAC. NAC response assessment was made with repeat cystoscopy and bladder tumour site biopsy within 21 days of completing NAC. Radiological evaluation with CT and/or MRI (qualitative interpretation) was also used to support clinical decision-making for patients with extra-vesical or nodal involvement. Conventional local response was assessment dichotomised into response (<T2) and poor response (≥T2) (**Supplementary Table S1**) (30).

Definitive radical treatment decision following NAC, i.e., radical cystectomy or chemo-radiotherapy to the bladder +/− pelvic lymph nodes was made as per standard clinical practice in accordance with national guidelines based on specialist urology multi-disciplinary team review (4).

### MRI protocol

Initial MRI scan was performed following TURBT and prior to cycle 1 NAC. MRI was repeated within 14 days of completing NAC chemotherapy and prior to scheduled cystoscopy and tumour site biopsy.

All patients were examined on a 1.5-T MR scanner using a four-channel sensitivity encoding body coil for anatomic coverage of the pelvis. Bladder distention was achieved by instructing patient to void, drink 350 ml water, and imaging 30 min later. Antispasmodic hyoscine butyl bromide (20mg) was administered intramuscularly before image acquisition to reduce motion artefact from bowel peristalsis (31).

Morphological imaging was performed with axial T1-weighted (T1W) and T2-weighted (T2W) sequences. DWI imaging was performed with an axial free-breathing single-shot echo-planar technique with parallel imaging and spectral adiabatic inversion-recovery fat suppression. Diffusion gradients with b-values of 0, 100, 150, 250, 500, and 750 s/mm^2^ were applied in three orthogonal directions.

Three different 1.5-T systems were commissioned over the study recruitment period and included Intera (Philips, Best, Netherlands) between 2007 and 2011, Avanto (Siemens Medical Systems, Erlangen, Germany) from 2011 to present, and Aera (Siemens Medical Systems, Erlangen, Germany) from 2011 to present. Aggregated analysis between the systems was deemed acceptable from phantom-based quality assurance testing (personal communication). All patients scanned on one vendor-specific scanner were re-imaged on the same scanner wherever possible. Imaging parameters used are summarised in **Supplementary Table S2**. Inter-scanning comparison is provided in the **Supplementary Material**.

### DWI evaluation and ADC analysis

DWI analysis was performed on masked data by a single investigator (SH) blinded to the pathological response using dedicated IDL-based software (ADEPT; The Institute of Cancer Research, London, UK). A region of interest (ROI) within the bladder was delineated using a computer-assisted segmentation technique identifying the area of greatest impeded diffusion on b750s/mm^2^ DWI corresponding to the tumour. The ROI was delineated on every b750s/mm^2^ DWI slice where the lesion appeared. Multi-focal tumours within the bladder were planned to be contoured individually given the possibility of a differential lesion response to NAC.

ROI drawn on the 750 s/mm^2^ image was transferred to the corresponding ADC map to calculate ADC at all six b-values (0, 50, 100, 250, 500, and 750 s/mm^2^), i.e., ADC_all_, and high b-values (b100, 250, 500, and 750 s/mm^2^), i.e., ADC_b100_, which is perfusion insensitive. This was calculated by fitting pixel-wise mono-exponential model using the following equation, S=S0 e^−b.ADC^, where S is the averaged signal intensity and S0 is the signal without diffusion weighting.

Individual pixel ADC values through the entire ROI for both ADC_all_ and ADC_b100_ was calculated. The mean, 10th, 25th, 50th, 75th, and 90th percentiles, skew, and kurtosis parameters for ADC_all_ and ADC_b100_ were derived from histogram (bin width, 1×10^−6^ mm^2^/s) analysis.

### Study objective

The study objective was to determine the association of pre-treatment ADC, post-treatment ADC, ΔADC, and %ΔADC with conventional tumour response and long-term survival outcomes in patients receiving NAC.

### Statistical considerations

Evaluable patients were defined as those who had baseline and response MRI acquired in accordance with the protocol and received at least one cycle of NAC. If no abnormal DWI signal was measurable on baseline MRI, i.e., patient had achieved complete functional response following TURBT, the proposed analysis was not possible, and the patient was deemed non-evaluable.

Patient characteristics were reported using descriptive statistics. Statistical analysis was performed using the above ADC histogram-derived features at ADC_all_ and ADC_b100_. Absolute change in ADC (ΔADC) following treatment was calculated as the difference between post-treatment and baseline ADC. The relative percentage change in (%ΔADC) was also calculated as %ΔADC = (post-NAC ADC – pre-NAC ADC)/pre-NAC ADC × 100.

The association between conventional treatment response with pre-treatment ADC, post-treatment ADC, ΔADC, and %ΔADC was analysed using non-parametric tests (Mann–Whitney U and Wilcoxon two sample tests). Where comparison between multiple groups was performed, Kruskal–Wallis analysis was used. Significance values were adjusted for multiple testing (Bonferroni correction).

Receiver operating characteristic (ROC) curve analysis was used to identify threshold predictive of treatment response with the highest specificity. ROC analysis for response status was performed using mean ADC, percentile, skew, and kurtosis for values derived from baseline image, post-treatment image, ΔADC, and %ΔADC. The parameter associated with an area under the curve (AUC) >0.6 and p <0.05 was considered significant.

ROC curve cut-point values for significant parameters was found by applying the Youden index *J*, defined as: *J* = max (sensitivity*c* + specificity*c* − 1), where *c* ranges over all possible criterion values. The sensitivity, specificity, positive predictive value (PPV), and negative predictive value (NPV) of the cut point were determined.

Median overall survival (OS), bladder-cancer-specific survival (bCSS), progression-free survival (PFS), and time to cystectomy were estimated using the Kaplan–Meier method. OS was defined as time from date of NAC start to date of death from any cause. bCSS was defined from NAC start to death due to bladder cancer; patients who died from other or unverified causes were censored at death. PFS was defined as the interval from NAC initiation to disease relapse not amenable to radical salvage. Time to cystectomy was defined from NAC start to date of operation. Surviving patients, progression free, and those with an intact bladder or lost to follow-up were censored at last follow-up.

Significant AUC cut points were used to dichotomise the patient population to determine the potential relationship with long-term endpoints. A comparison between the curves was made with log-rank test. Analyses were carried using SPSS v.22 (IBM, Chicago, Ill., USA) and MedCalc Version 16.4.3 (Medcalc software Ltd, Ostend, Belgium).

## Results

### Patient characteristics

**Figure 1:**
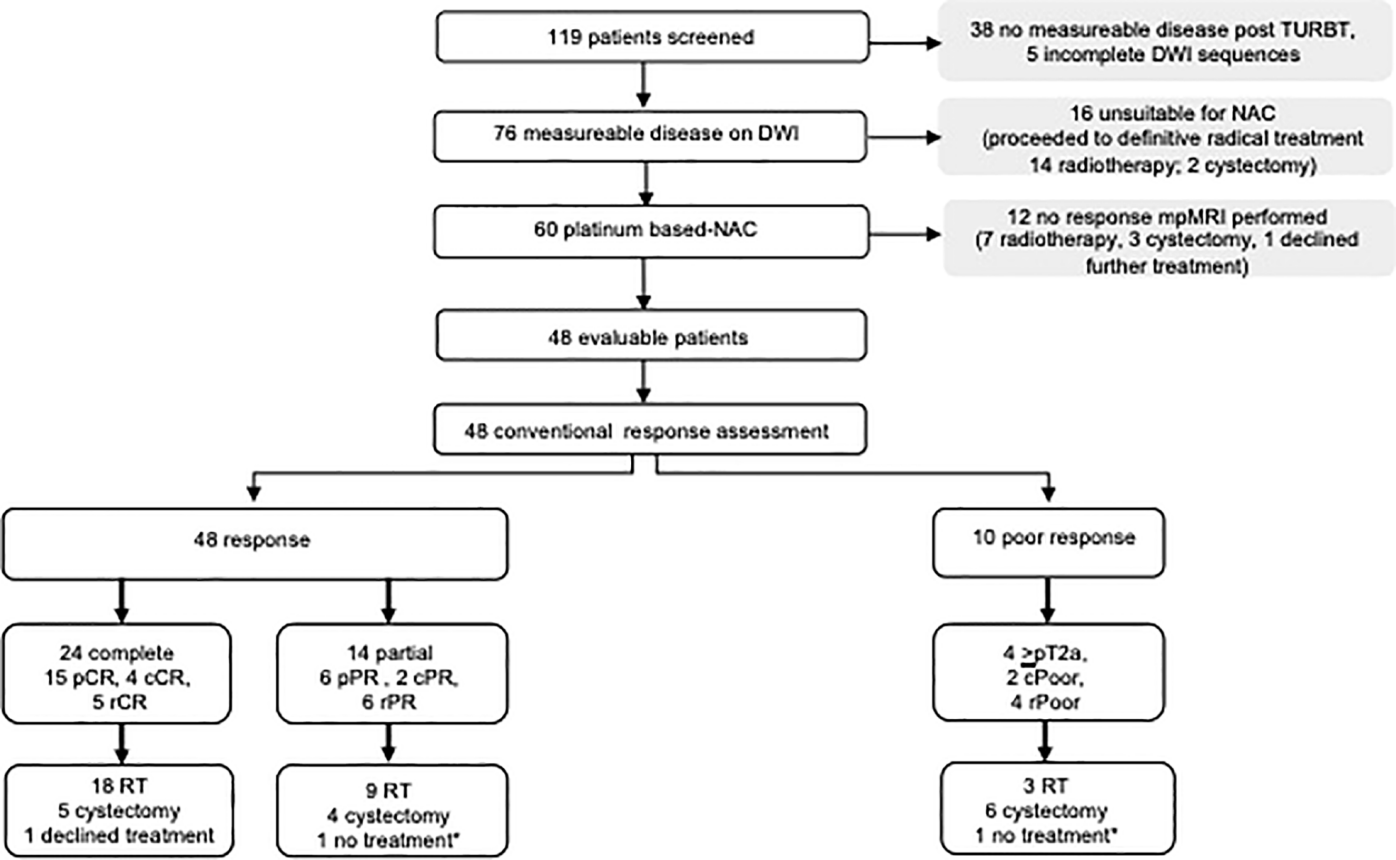
Patient study flow. NAC, neo-adJuvant chemotherapy; DWI, diffusion weighted MRl scan; pCR, pathological complete response; cCR, clinical complete response on cystoscopy; pPR, pathological partial response; rCR, radiological complete response; rPR, radiological partial response; cPoor, clinical poor resposnse on cystoscopy; rPoor, radiological poor response, RT-radiotherapy. *death pnor to definitive radical treatment due to cause unrelated to bladder cancer or NAC.

Between September 2007 and August 2013, 119 patients were screened. Forty-eight patients (40%) were available for analysis. Thirty-eight patients (32%) who had no visible abnormality on DWI post TURBT, 17 patients (14%) who had MRI acquisition that deviated from the protocol, and 16 (13%) who proceeded directly to radical treatment without NAC were excluded. Patient study flow is shown in **Figure 1**.

Patient and tumour characteristics are presented in **Table 1**. All patients had predominant urothelial histology and unifocal bladder tumours. The median time from diagnostic TURBT to baseline MRI scan was 35 days (range, 8–110 days). A total of 85.4% (41/48) of the patients completed NAC as planned. Cystoscopic evaluation of NAC response was performed in 69% (33/48) of the patients; 52% (25/48) also underwent tumour site biopsy. The remaining patients (31%, 15/48) had radiological NAC response assessment only. Seventy-nine percent (38/48) of the patients demonstrated NAC response.

**Table 1:** Patient and tumour characteristics.

Age	Median years of 69.5 (range, 41–84)
**Gender**	
Male	40
Female	8
**Stage of the primary**	
T2	21
T3	21
T4	6
**Highest stage**	
T2	20
T3	17
T4	5
N+	6
**Chemotherapy protocol**	
Gemcitabine–Cisplatin*	42
Gemcitabine–Carboplatin	6
Number of cycles (median)**	4 (range 1–6)
**Conventional assessment**	
Cystoscopy ± biopsy	33
Radiology alone	15
**Conventional response**	
Complete	24
Partial	14
Poor	10
**Definitive treatment**	
Radiotherapy (with radiosensitisation)^+^	30 (18)
Cystectomy	15
No further treatment following neo-adjuvant chemotherapy	3

NAC, neo-adjuvant chemotherapy.

*Three patients started gemcitabine–cisplatin but were switched to gemcitabine–carboplatin, two because of renal impairment and one because of cardiac co-morbidity.

**Forty-one of the 48 patients completed their planned course of NAC.

One patient stopped after cycle 1 because of symptomatic disease progression, one patient stopped at cycle 1.5 (out of planned three cycles) following a stroke, one patient stopped at cycle 2 (out of planned three cycles) because of duodenal perforation, one patient stopped at cycle 3 (out of planned six) because of poor interval response, one patient stopped at cycle 5 (out of planned six cycles) because of deteriorating renal function, one patient stopped at cycle 5 (out of planned six cycles) because of patient choice, one patient stopped at cycle 5 (out of planned six cycles) because death due to cause unrelated to disease or treatment.

^+^Eighteen patient received radiosensitisation with concurrent chemotherapy (17 patients received mitomycin C and 5FU; 1 patient received gemcitabine).

### Response evaluation using DWI change

All tumours seen on T2W were also identified on b750s/mm^2^ DWI. The median DWI bladder lesion volume prior to NAC was 7.62 cm^3^ (range, 0.34–139.92). Median baseline tumour ADC_all_ mean and ADC_b100_ mean were 1.35×10^−3^ mm^2^/s (range, 0.85–2.04×10^−3^) and 1.22×10^−3^ mm^2^/s (range 0.78-1.86x10^-3^) (p<0.001) respectively.

**Figure 2:**
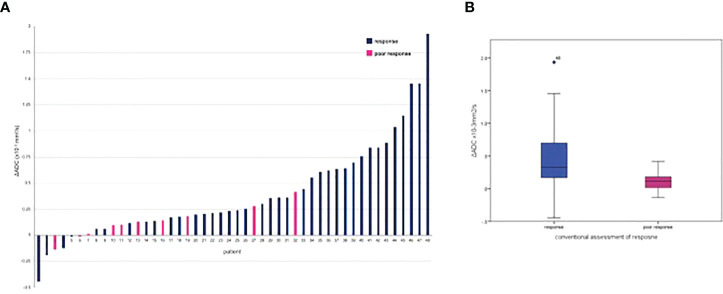
ΔADC_all_ mean following neo-adjuvant chemotherapy grouped by conventional response: **(A)** per patient waterflow plot; **(B)** box blot.

ADC histogram characteristics calculated from baseline and post-NAC at ADC_all_ and ADC_b100_ are presented in **Supplementary Table S3**. ΔADC and %ΔADC grouped by response is given in **Table 2**. **Figure 2** summaries ΔADC seen in response groups.

**Table 2:** Change in ADC histogram parameters to neo-adjuvant chemotherapy as grouped by conventional response assessment.

Parameter	ΔADC							%ΔADC change						
	Response (n = 38)			Poor response (n = 10)			p value*	Response (n = 38)			Poor response (n = 10)			p value*
	Median	Min	Max	Median	Min	Max		Median	Min	Max	Median	Min	Max	
**ΔADC_all_**														
Mean	0.32	−0.44	1.93	0.11	−0.14	0.48	0.009	21.7	−24.83	197.42	8.23	−12.96	37.11	0.013
10^th^ percentile	0.12	−1.22	1.00	0.06	−0.08	0.35	0.793	10.98	−100.00	141.06	7.81	−9.94	44.7	0.793
25^th^ percentile	0.21	−1.51	0.99	0.04	−0.06	0.32	0.114	18.49	−100.00	137.75	4.39	−7.26	34.68	0.114
50^th^ percentile	0.27	−0.42	1.61	0.06	−0.07	0.34	0.008	21.85	−25.96	183.01	4.84	−7.1	31.49	0.01
75^th^ percentile	0.44	−0.61	2.44	0.14	−0.11	0.49	0.002	29.68	−28.62	223.23	10.32	−9.81	31.27	0.002
90^th^ percentile	0.65	−0.72	3.66	0.18	−0.32	0.68	0.005	37.46	−24.92	247.60	10.60	−21.55	42.89	0.011
Skew	−0.55	−9.11	2.21	−0.07	−0.97	6.42	0.077	−36.23	−194.97	772.82	−4.34	−44.74	224.52	0.035
Kurtosis	−1.67	−182.2	7.79	0.555	−0.13	1.36	0.077	−72.74	−425.82	1,685.70	12.43	−93.82	1518.58	0.045
**ΔADC_b100_**														
Mean	0.30	−0.15	1.42	0.09	−0.21	0.045	0.007	25.64	−10.95	147.82	7.59	−21.29	43.00	0.016
10^th^ percentile	0.09	−1.28	0.83	0.05	−0.16	0.25	0.698	9.60	−100.00	137.50	5.33	−24.06	32.6	0.698
25^th^ percentile	0.12	−1.54	0.89	0.05	−0.14	0.33	0.232	12.61	−100.00	160.99	6.15	−17.44	38.68	0.222
50^th^ percentile	0.28	−0.17	1.09	0.06	−0.11	0.37	0.021	25.64	−10.95	147.83	5.92	−12.61	37.44	0.037
75^th^ percentile	0.54	−0.21	1.98	0.12	−0.14	0.52	<0.0001	34.84	−13.78	221.66	10.1	−14.03	44.25	0.002
90^th^ percentile	0.67	−0.32	2.44	0.17	−0.32	0.80	0.008	35.12	−17.35	208.73	11.34	−23.89	57.66	0.021
Skew	0.02	−3.35	1.19	−0.02	−2.48	0.78	0.930	−36.28	−5788.78	1,525.54	−2.23	−82.53	77.80	0.360
Kurtosis	−0.57	−50.64	4.28	−0.49	−10.39	3.14	0.608	−84.71	−1840.56	529.86	−15.60	−83.62	160.78	0.077

*Independent sample Mann Whitney U test, p values refer to comparison between response and poor response groups.

Negative values reflect a decrease from baseline. Mean and percentile ADC are given in x10^-3^ mm^2^/s. Skew and kurtosis are absolute values.

The ΔADC mean was significantly greater in those achieving response with NAC compared to poor response at ΔADC_all_ (0.32 × 10^−3^ mm^2^/s versus 0.11 × 10^−3^ mm^2^/s; p=0.009) and ΔADC_b100_ (0.30 × 10^−3^ versus 0.09 × 10^−3^ mm^2^/s; p=0.007). A 21.70% increase in %ΔADC_all_ mean was seen in response compared to the 8.23% increase in poor response (p=0.013). This corresponded to a %ΔADC_b100_ mean increase of 25.64% in response versus 7.59% for poor response (p=0.016).

The individual AUC values calculated at baseline ADC, post-NAC ADC, ΔADC, and %ΔADC in histogram characteristics at ADC_all_ and ADC_b100_ are presented in **Supplementary Table S4**. The corresponding significant AUC cut-off criteria with associated sensitivity, specificity, PPV, and NPV are presented in **Table 3**.

**Table 3:** Predictive power of neo-adjuvant chemotherapy response derived from significant ADC cut point parameters.

ADC parameter	ADC_all_									ADC_b100_								
	*Cut point	Sensitivity	95% CI	Specificity	95% CI	PPV	95% CI	NPV	95% CI	*Cut point	Sensitivity	95% CI	Specificity	95% CI	PPV	95% CI	NPV	95% CI
**Post NAC ADC (x10^-3^ mm^2^/s)**																		
**Mean**	1.65	65.8	48.6 - 80.4	90.0	55.5 - 99.7	96.2	80.4 - 99.9	40.9	20.7 - 63.6	1.61	55.3	38.3 - 71.4	100.0	69.2 - 100.0	100.0	83.9 - 100.0	37.0	19.4 - 57.6
**50th percentile**	1.54	55.3	38.3 -71.4	90.0	55.5 - 99.7	95.5	77.2 - 99.9	34.6	17.2 - 55.7	1.50	55.3	38.3 - 71.4	100.0	69.2 - 100.0	100.0	83.9 - 100.0	37.0	19.4 - 57.6
**75th percentile**	1.63	86.8	71.9 - 95.6	70.0	34.8 - 93.3	91.7	77.5 - 98.2	58.3	27.7 - 84.8	1.79	57.9	40.8 - 73.7	100.0	69.2 - 100.0	100.0	84.6 - 100.0	38.5	20.2 - 59.4
**90th percentile**	2.20	68.4	51.3 - 82.5	90.0	55.5 - 99.7	96.3	81.0 - 99.9	42.9	21.8 - 66.0	2.30	55.3	38.3 - 71.4	100.0	69.2 - 100.0	100.0	83.9 - 100.0	37.0	19.4 - 57.6
**Absolute change in ADC (x10^-3^ mm^2^/s)**																		
**Mean**	0.18	71.1	54.1-84.6	80.0	44.4 - 97.5	93.1	77.2 - 99.2	42.1	20.3 - 66.5	0.19	65.8	48.6 - 80.4	90.0	55.5 - 99.7	96.2	80.4 - 99.9	40.9	20.7 - 63.6
**50th percentile**	0.18	65.8	48.6 - 80.4	90.0	55.5 - 99.7	96.2	80.4 - 99.9	40.9	20.7 - 63.6	0.11	68.4	51.3 - 82.5	80.0	44.4 - 97.5	92.9	76.5 - 99.1	40.0	19.1 - 63.9
**75th percentile**	0.17	84.2	68.7 - 94.0	80.0	44.4 - 97.5	94.1	80.3 - 99.3	57.1	28.9 - 82.3	0.22	79.0	62.7 - 90.4	90.0	55.5 - 99.7	96.8	83.3 - 99.9	52.9	27.8 - 77.0
**90th percentile**	0.68	50.0	33.4 - 66.6	100.0	69.2 - 100.0	100.0	82.4 - 100.0	34.5	17.9 - 54.3	0.50	60.5	43.4 - 76.0	90.0	55.5 - 99.7	95.8	78.9 - 99.9	37.5	18.8 - 59.4
**Relative percentage change in ADC**																		
**Mean**	18.0	57.9	40.8 - 73.7	90.0	55.5 - 99.7	95.7	78.1 - 99.9	36.0	18.0 - 57.5	18.1	60.5	43.4 - 76.0	90.0	55.5 - 99.7	95.8	78.9 - 99.9	37.5	18.8 - 59.4
**50th percentile**	16.5	60.5	43.4 - 76.0	90.0	55.5 - 99.7	95.8	78.9 - 99.9	37.5	18.8 - 59.4	8.3	68.4	51.3 - 82.5	80.0	44.4 - 97.5	92.9	76.5 - 99.1	40.0	19.1 - 63.9
**75th percentile**	13.4	81.6	65.7 - 92.3	80.0	44.4 - 97.5	93.9	79.8 - 99.3	53.3	26.6 - 78.7	15.5	73.7	56.9 - 86.6	90.0	55.5 - 99.7	96.6	82.2 - 99.9	47.4	24.4 - 71.1

*Only cut points for significant AUC presented. The discriminatory threshold is greater than the cut point determined. NPV, negative predictive value; PPV, positive predictive value.

No baseline ADC parameter was significantly associated with NAC response. Significant AUC values associated with NAC response was identified at both ADC_all_ and ADC_b100_ for ADC mean, 50th, 75th, and 90th percentiles of post-NAC ADC and ΔADC, and at ADC mean, 50th, and 75th percentile of %ΔADC. The parameter that had the highest specificity in discriminating between NAC response and poor response was 75th percentile ADC (AUC, 0.8; p=0.01) (**Supplementary Table S4**).

ΔADC_all_ mean of 0.18 × 10^−3^mm^2^/s and ΔADC_b100_ mean of 0.19 × 10^−3^mm^2^/s, respectively, predicted NAC response with sensitivity of 71.1% and 65.8%, specificity of 80.0% and 90.0%, PPV of 93.1% and 96.2%, and NPV of 42.1% and 40.9%, respectively.

%ΔADC_all_ mean and %ΔADC_b100_ mean of 18.0% and 18.1%, respectively, predicted NAC response with sensitivity of 57.9% and 60.5%, specificity of 90.0% and 90.0%, PPV 95.7% and 95.8%, and NPV 36.0% and 37.5%, respectively.

The 75th percentile ΔADC_all_ of 0.17 × 10^−3^ mm^2^/s and %ΔADC_all_ of 13.4% predicted NAC response with sensitivity of 84.2% and 81.6%; specificity of 80.0% and 80.0%, PPV of 94.1% and 93.9%, and NPV of 57.1% and 53.3% respectively. The 75th percentile ΔADC_b100_ of 0.22 × 10^−3^ mm^2^/s and %ΔADC _b100_ of 15.5% predicted NAC response with sensitivity of 79.0% and 68.4%, specificity of 90.0% and 90.0%, PPV of 96.8% and 96.6%, and NPV 52.9% and 47.4%, respectively.

### DWI as biomarker of long-term outcome

After median follow-up of 103 months (95% CI, 63.9–141.7), 31.3% (15/48) of the patients were alive and disease free. Of the 33 patients who died, 42.4% (14/33) of deaths were attributed to bladder cancer, 30.3% (10/33) were due to other causes, and 27.3% (9/33) deaths were unknown/unverified. Median OS and time to cystectomy was 31 months (95% CI, 18.5–86.9) and 44 months (95% CI, 10.0–76.2), respectively. Overall median PFS was not assessable using the Kaplan–Meier method.

**Figure 3:**
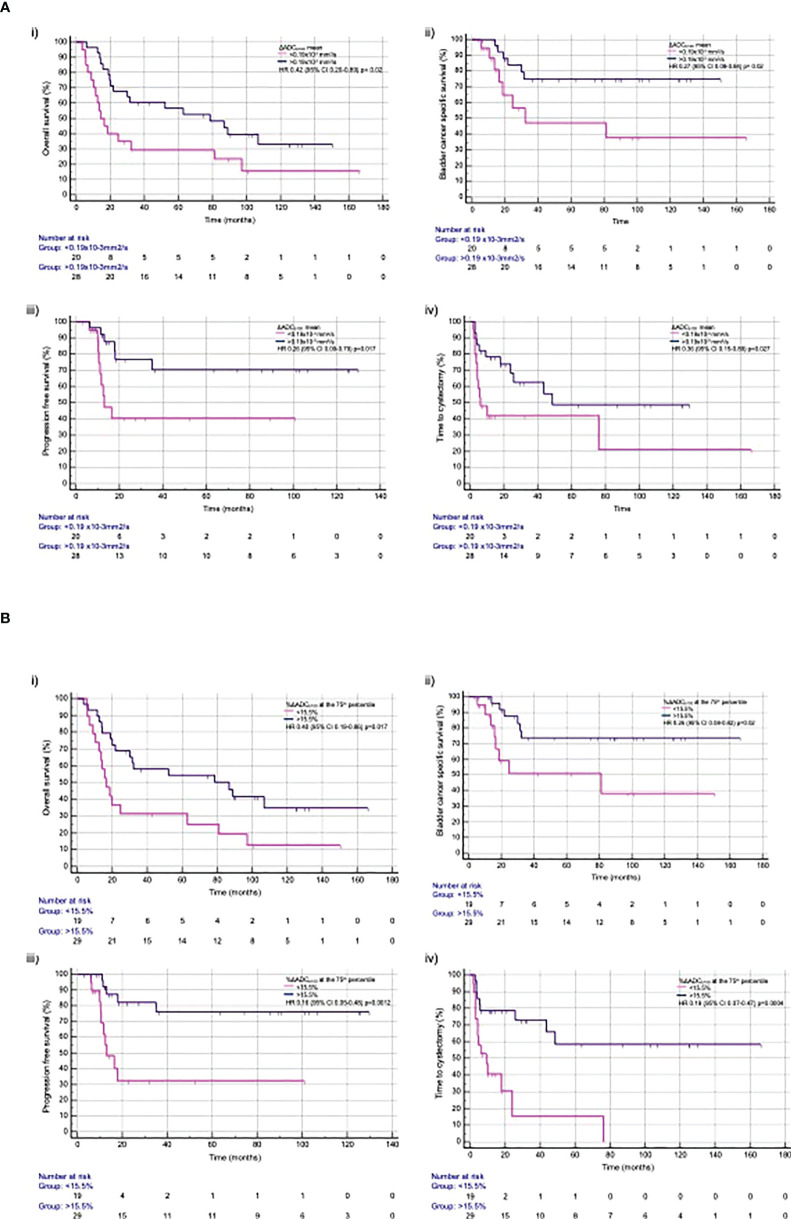
Time to event outcomes with **(A)** ΔADC_b100_ mean and **(B)** %ΔADC_b100_ at the 75th percentile for **(i)** overall survival, **(ii)** bladder cancer specific free survival, **(iii)** progression-free survival, and **(iv)** time to cystectomy.

ΔADC_b100_ mean >0.19×10^−3^ mm^2^/s was associated with a significantly improved OS (HR, 0.42; 95% CI, 0.20–0.89; p=0.023), bCSS [HR, 0.27 (95% CI, 0.08–0.84; p=0.0240)], PFS (HR, 0.26; 95% CI, 0.09–0.79; p=0.017), and time to cystectomy (HR, 0.36; 95% CI, 0.15–0.89; p=0.0271) (**Figure 3**).

%ΔADC_b100_ >15.5% at 75th percentile was associated with significant improvement in OS (HR, 0.40; 95% CI, 0.19–0.86; p=0.0179), bCSS (HR, 0.26; 95% CI, 0.08–0.82; p=0.0214), PFS (HR, 0.16; 95% CI, 0.05–0.48; p=0.0012), and time to cystectomy (HR, 0.19; 95% CI, 0.07–0.47; p=0.0004) (**Figure 3**). A summary of all time to event outcomes according to the derived imaging biomarkers is given in **Supplementary Table S5**.

## Discussion

Risk stratification is critical for personalising MIBC follow-up schedules, safely selecting candidates for bladder preservation protocols, and identifying poor prognosis disease who could benefit from treatment intensification following NAC. We presented first quantitative DWI analysis to both successfully determine NAC response and survival outcomes in localised MIBC. It provides supporting evidence that imaging biomarkers could be non-invasive tools for NAC assessment and means of determining NAC sensitivity. Quantitative imaging biomarkers may therefore provide adjunct to molecular predictors currently being investigated (8).

Liquid-biopsy-derived biomarkers from plasma or urine do not present threat to imaging biomarkers but are likely to compliment predictive and prognostic power (8, 32–34). Circulating tumour DNA (ctDNA) can be detected in most patients with metastatic bladder but has attenuated sensitivity in those with localised organ-confined disease. ctDNA bladder cancer detection rates fall from 73% in metastatic disease to 14% in organ-confined disease (35–37). However, ctDNA presence prior to NAC, dynamics during NAC, and detection at follow-up provide prognostic information (38). The detection of urinary bladder cancer associated mutations has been evaluated in the context of diagnosis and non-muscle-invasive bladder cancer surveillance (39, 40). Their role in predicting NAC response is yet to be determined.

A perceived study drawback may be that NAC response was not defined pathologically in all our patients. Biopsy or TURBT was not always undertaken if there was obvious presence or absence of tumour at cystoscopy that would not have altered the patient’s preferred definitive treatment choice. When performed, its limitation in diagnostic accuracy to assess true residual disease following NAC is acknowledged (41). In a series of 114 patients who underwent TURBT following NAC and then proceeded to radical cystectomy, Becker et al. demonstrated that 32% of patients were falsely downstaged as compared to their final pathology, which demonstrated residual MIBC (≥ypT2). Subset analysis of this series demonstrated that 81% (17/21) who had no residual disease visible on cystoscopy were T0 at TURBT, with 24% (4/21) having ≥ypT2 at cystectomy (41). It is also recognised that pCR remains a surrogate endpoint with limitations when evaluating neoadjuvant therapies in MIBC (42). Arguably, we have therefore presented a more direct measure of clinical relevance with the corresponding time to survival event endpoint analyses.

The study protocol precedes VI-RADS image acquisition recommendation (17). Nevertheless, many of the technical considerations made therein were used in the study protocol, as the authors contributed to VI-RADS development. VI-RADS mpMRI qualitative scoring is used to aid pre-treatment staging, but its application on the assessment of treatment response is yet to be determined.

Qualitative DWI response reporting appears reliant on expertise. Although inter-reader agreement is good in the diagnostic setting (43), identifying complete treatment response is subject to greater reporting variation (44). Quantitative image analysis, therefore, has the potential to overcome reporting discrepancy.

ADC was calculated and presented using all b-values of 0–750 s/mm^2^ (ADC_all_) and high b-values of 100–750 s/mm^2^ (ADC_b100_). Biophysical processes such as blood flow and perfusion can increase apparent water mobility and adversely impact on accurately measuring tissue water diffusion. The use of high b-values can suppress these contributing effects (45). The significant difference seen at baseline between mean ADC_all_ and mean ADC_b100_ illustrates the magnitude of perfusion effects and reflects the intrinsic vascularity of bladder tumours (28). It highlights the importance of selecting appropriate b-values for ADC calculation and for quantitative DWI biomarker development in these circumstances.

Given that ADC estimates depend on the chosen b-values, direct comparison of absolute ADC values between studies with differing acquisition protocols should be avoided (28). Similarly, the specific ADC thresholds defined in this study relate to the decreasing cellular density associated with NAC response and are unlikely to be directly applicable to assess response with other treatment modalities. For example, stromal tissue effector cell infiltration seen with immunotherapy response is cited as the potential reason why quantitative ADC assessment was unreliable in assessing neoadjuvant pembrolizumab response within the PURE-01 study (44).

No measured pre-treatment baseline ADC feature could successfully identify NAC outcome. A potential confounding factor is the time variation from initial diagnostic TURBT to baseline MRI, as the degree of post-procedural inflammation and oedema could have impacted on the diffusion characteristics of the absolute baseline ADC reading (28). Repeatability of baseline ADC is estimated to be approximately 15%; therefore, any increase following treatment greater than this can be considered real (46). It is therefore expected that the ADC difference between good tumour response and poor response is larger than the variation caused by inflammation, oedema, and fibrosis caused by artefact post-TURBT.

Another likely contributing factor impacting on the predictive potential of the baseline MRI is vendor variation; 54.2% (26/48) of baseline MRI scans were performed on the Intera system, 39.6% (19/48) on the Aera system, and 6.3% (3/48) on the Avanto system. *Post-hoc* analysis provided in **Supplementary Material 1** identified that the baseline mean ADC calculation was statistically significant between the Intera and Aera systems. Importantly, however, only three patients had a scanner/vendor switch at their post-NAC MRI. Therefore, individual patients provided internal control of this variable when ΔADC was considered. The measured ΔADC between the vendors was not statistically significant, and the ΔADC was large enough to mitigate scanner differences.

Although no baseline ADC characteristic could determine NAC response, scanning earlier during NAC may help provide this information. A feasibility study of 16 patients demonstrated change in mean ADC after cycles 1 and 2 of NAC-predicted final response (46). Early prediction of final chemotherapy response has also been demonstrated after two cycles in those with more advanced disease using DCE (47). We have expanded our current protocol to include scanning following cycle 1 to determine whether earlier quantitative signal can be successfully identified.

Additional predictive potential may come from considering alternative DWI analysis. In this study, ADC was derived by fitting a mono-exponential function. However, in other tumours, this model does not fully describe the DWI signal (48). Alternative models have been applied to better describe tumour behaviour (49–51). Radiomic feature extraction from the MRI data may also add to predictive power (52). Textural features derived from DWI and ADC maps can predict bladder tumour grade and muscle-invasive disease (53–55). Its application in bladder cancer treatment response prediction remains novel.

A proportion of the patients were deemed non-evaluable at baseline because no identifiable DWI signal was seen at initial scan following TURBT. In the current standard MIBC diagnostic paradigm wedded to TURBT for T staging, universal utility of an imaging biomarker to predict NAC response is likely to be limited. The BladderPath Trial (ISRCTN 35296862) will help determine safety of an mpMRI-directed diagnostic pathway in MIBC (56). It is anticipated that this study will inform if patients with high likelihood of MIBC based on mpMRI and tumour biopsy alone can safely proceed to treatment without TURBT. If successful, it would make DWI-derived biomarker to assess treatment response more widely applicable.

Before the defined DWI biomarkers can be tested within clinical trials to inform treatment decision-making, technical validation to assess reproducibility and repeatability in the multi-centre setting is required (9). This work is planned. We envisaged that quantitative DWI biomarkers will be used in the future as part of the qualifying criteria for bladder preservation following NAC alongside various DDR mutations and cell cycle and regulatory genes that are currently being evaluated in prospective studies.

## Conclusion

We present first quantitative DWI analysis to both successfully determine NAC response and provide prognostic information regarding long-term clinical MIBC outcomes following NAC. Multi-centre validation to assess reproducibility and repeatability is required before testing within clinical trials to inform MIBC treatment decision-making.

## Data availability statement

The datasets presented in this article are not readily available. The Data and Sample Access Policy is available on our external facing web pages. Priority will be given to projects from within the original study proposal and access will usually be reserved for study purposes until those studies are concluded. Data and/or samples will not be released where this could impact on the reporting of on-going research questions of the study. Requests to access the datasets should be directed to shaista.hafeez@icr.ac.uk.

## Ethics statement

This study was reviewed and approved by The Royal Marsden NHS Foundation Trust. The patients/participants provided their written informed consent to participate in this study.

## Author contributions

All authors meet at least one the criteria recommended by the IJCME. SH performed the analysis and wrote the first draft of the manuscript. All authors contributed to the article and approved the submitted version.

## Funding

The authors acknowledge this study represents independent research supported by the National Institute for Health Research (NIHR) Biomedical Research Centre at The Royal Marsden NHS Foundation Trust and The Institute of Cancer Research, London. The views expressed are those of the author(s) and not necessarily those of the NIHR or the Department of Health and Social Care. The work was supported by Cancer Research UK programme grant (C33589/A19727 and C33589/A28284).

## Conflict of interest

The authors declare that the research was conducted in the absence of any commercial or financial relationships that could be construed as a potential conflict of interest.

## Publisher’s note

All claims expressed in this article are solely those of the authors and do not necessarily represent those of their affiliated organizations, or those of the publisher, the editors and the reviewers. Any product that may be evaluated in this article, or claim that may be made by its manufacturer, is not guaranteed or endorsed by the publisher.
