# Supplementary material for: Diffusion-weighted MRI to determine response and long-term clinical outcomes in muscle-invasive bladder cancer following neoadjuvant chemotherapy
Source: Front Oncol. 2022 Nov 14;12:961393. doi: 10.3389/fonc.2022.961393 (PMC9702046; doi:10.3389/fonc.2022.961393)
Supplement: Supplementary file 1 [file DataSheet_1.pdf]

Supplementary Material

Table.1 Categorisation of NAC response based on conventional assessment

Response status		Pathological	Clinical (Endoscopic, and or EUA)	Radiological (CT, and or MRI)
**Response**	Complete	pCR (T0)	cCR	rCR
	Partial	pPR (≤T1)	cPR	rPR
**Poor response**	Poor	≥T2	No response, or evidence of clinical progression	PD, or persistent ≥T2

Abbreviations; pCR pathological complete response, pPR pathological partial response, cCR clinical complete response, rCR radiological complete response, rPR radiological partial response, PD disease progression

Supplementary Material

Table 2 . Summary of imaging protocol used

*Parameter			
1.5T MRI system	Intera; Philips Healthcare, Best, Netherlands	Avanto; Siemens Healthcare, Erlangen, Germany	Aera; Siemens Healthcare, Erlangen, Germany
Pulse sequence	single- shot, echo planar imaging	single- shot, echo planar imaging	single- shot, echo planar imaging
TE/TR (ms)	62/3637	72/3200	72/4400
Number of averages	3	4	3
FOV (mm)	400x400	219x178	219x184
FOV technique	fFOV	sFOV	sFOV
Acquisition matrix	112x110	138x112	138x116
Reconstruction matrix	256x256	276x224	138x116
Slice thickness (mm)	7	5	5
Inter slice thickness (distance factor)	0.7	0	0
Parallel imaging	SENSE	ISOTROPIC	GRAPPA
Orientation	Axial	Axial	Axial
Phase encode direction	AP	AP	AP
Receiver bandwidth (Hz per pixel)	1880	1725	1295
b values (s/mm^2)	0, 50,100,250,500 and 750	0, 50,100,250,500 and 750	0, 50,100,250,500 and 750
Total number and directions of diffusion sensitising gradients	Trace-weighted images 3	Trace-weighted images 3	Trace-weighted images 3
Fat suppression	SPAIR	SPAIR	SPAIR

*The total MR acquisition protocol is 35 minutes of which the DWI acquisition time was approximately 7 minutes.

Abbreviations T, Tesla field strength; TE Time to echo; TR repetition time; FOV field of view

Supplementary Material

Table 3. ADC histogram parameters in responders and non-responders to neo-adjuvant chemotherapy

Parameter	Response (n=38)							Poor response (n=10)						
	Baseline ADC			Post NAC ADC			p value*	Baseline ADC			Post NAC ADC			p value*
	Median	Min	Max	Median	Min	Max		Median	Min	Max	Median	Min	Max	
**ADC_{all}**														
Mean	1.14	0.85	2.04	1.74	1.07	3.18	<0.0001	1.22	1.07	1.56	1.40	0.93	1.83	0.037
10th percentile	0.93	0.49	1.59	1.03	0.00	2.19	0.058	0.85	0.75	1.11	1.04	0.68	1.30	0.114
25th percentile	1.11	0.62	1.85	1.23	0.00	2.42	0.001	0.98	0.84	1.29	1.15	0.78	1.40	0.074
50th percentile	1.32	0.82	2.00	1.64	1.05	3.14	<0.0001	1.14	0.97	1.47	1.28	0.90	1.54	0.037
75th percentile	1.60	0.92	2.28	2.19	1.26	4.01	<0.0001	1.35	1.15	1.75	1.52	1.04	2.24	0.028
90th percentile	1.97	1.05	2.97	2.86	1.63	5.68	<0.0001	1.79	1.50	2.20	2.05	1.19	2.74	0.047
Skew	1.36	-0.25	11.80	0.93	-0.83	4.09	0.005	1.85	1.11	2.86	1.69	1.05	9.29	0.959
Kurtosis	3.20	-0.93	194.78	1.16	-1.39	21.61	0.002	5.21	1.77	13.52	4.71	0.84	1.45	0.575
**ADC_{b100}**														
Mean	1.23	0.78	1.86	1.67	0.93	2.91	<0.0001	1.09	1.00	1.40	1.25	0.78	1.60	0.059
10th percentile	0.82	0.33	1.34	0.90	0.00	2.05	0.126	0.74	0.64	1.05	0.91	0.54	1.27	0.093
25th percentile	0.98	0.56	1.58	1.11	0.00	2.32	0.001	0.88	0.79	1.20	1.04	0.65	1.35	0.059
50th percentile	1.20	0.73	1.90	1.57	0.83	2.79	<0.0001	1.03	0.89	1.38	1.18	0.78	1.50	0.037
75th percentile	1.43	0.86	2.29	2.12	1.27	3.33	<0.0001	1.24	1.05	1.57	1.40	0.91	1.79	0.022
90th percentile	1.77	0.99	2.66	2.54	1.52	4.39	<0.0001	1.55	1.38	1.86	1.81	1.05	2.30	0.047
Skew	0.79	-0.60	4.77	0.81	0.05	2.12	0.744	1.40	0.85	3.0	1.31	0.52	2.85	0.721
Kurtosis	1.30	-0.93	54.8	0.56	-1.53	6.27	0.112	3.30	1.61	12.42	2.64	0.35	11.99	0.878

*Wilcoxon signed rank test, p values refer to comparison between baseline pre chemotherapy and post chemotherapy in responding and non-responding groups.
Mean and percentiles ADC are given in $x10^{-3}$ mm^2/s. Skew and kurtosis are absolute values

Supplementary Material

Table 4. ROC analysis of ADC parameters

Parameter	Derived from ADC_{all} values				Derived from ADC_{b100}			
	AUC	*p value	95%CI		AUC	*p value	95%CI	
**Baseline ADC**								
Mean	0.56	0.57	0.39	0.74	0.55	0.66	0.37	0.73
10th percentile	0.46	0.71	0.26	0.66	0.47	0.78	0.27	0.67
25th percentile	0.48	0.85	0.29	0.67	0.49	0.93	0.30	0.68
50th percentile	0.59	0.44	0.42	0.76	0.58	0.47	0.41	0.76
75th percentile	0.61	0.35	0.44	0.77	0.59	0.45	0.41	0.77
90th percentile	0.60	0.40	0.42	0.77	0.62	0.30	0.45	0.78
Skew	0.40	0.37	0.22	0.57	0.34	0.16	0.18	0.50
Kurtosis	0.37	0.25	0.20	0.54	0.31	0.10	0.16	0.46
**Post NAC ADC**								
Mean	0.74	**0.04**	0.58	0.90	0.76	**0.02**	0.61	0.90
10th percentile	0.46	0.71	0.28	0.63	0.46	0.75	0.28	0.65
25th percentile	0.60	0.38	0.43	0.77	0.58	0.49	0.40	0.76
50th percentile	0.75	**0.03**	0.59	0.90	0.76	**0.02**	0.62	0.91
75th percentile	0.80	**0.01**	0.65	0.96	0.80	**0.01**	0.66	0.94
90th percentile	0.75	**0.03**	0.60	0.89	0.75	**0.03**	0.60	0.90
Skew	0.19	0.01	0.05	0.32	0.29	0.07	0.13	0.45
kurtosis	0.26	0.03	0.08	0.43	0.29	0.06	0.11	0.46
**Absolute ADC change**								
Mean	0.74	**0.04**	0.58	0.90	0.78	**0.02**	0.62	0.93
10th percentile	0.50	0.98	0.32	0.69	0.55	0.68	0.38	0.72
25th percentile	0.67	0.13	0.51	0.84	0.62	0.29	0.45	0.80
50th percentile	0.78	**0.01**	0.63	0.93	0.75	**0.03**	0.59	0.90
75th percentile	0.79	**0.01**	0.65	0.94	0.82	**0.01**	0.69	0.95
90th percentile	0.74	**0.04**	0.58	0.89	0.74	**0.04**	0.59	0.89
Skew	0.29	0.06	0.12	0.46	0.45	0.68	0.29	0.62
Kurtosis	0.33	0.13	0.10	0.56	0.42	0.49	0.23	0.62
**Relative percentage ADC change**								
Mean	0.73	**0.04**	0.57	0.89	0.75	**0.03**	0.58	0.92
10th percentile	0.51	0.95	0.32	0.69	0.55	0.66	0.38	0.72
25th percentile	0.67	0.14	0.50	0.84	0.63	0.26	0.45	0.80
50th percentile	0.77	**0.02**	0.62	0.92	0.73	**0.05**	0.55	0.90
75th percentile	0.79	**0.01**	0.64	0.94	0.79	**0.01**	0.65	0.94
90th percentile	0.71	0.06	0.55	0.87	0.71	0.07	0.55	0.88
Skew	0.27	0.04	0.11	0.42	0.31	0.10	0.16	0.47
Kurtosis	0.29	0.07	0.11	0.47	0.30	0.09	0.15	0.46

*Significant results are those with AUC >0.6 p<0.05

Supplementary Material

Table 5. Time to event outcomes according to the derived imaging biomarkers

Imaging biomarker	Time to event analysis			
	Overall survival	Bladder cancer specific survival	Progression free survival	Time to cystectomy
ΔADC_{all} mean. >0.18x10^{-3} mm^2/s	HR 0.71 (95% CI 0.34-1.49) p=0.37	HR 0.43 (95% CI 0.14-1.34) p=1.15	HR 0.58 (95% CI 0.20-1.69) p=0.32	HR 0.20 (95% CI 0.08-0.51) **p=0.0008**
ΔADC_{b100} mean >0.19x10^{-3} mm^2/s	HR 0.42 (95% CI 0.20-0.89) **p= 0.02**	HR 0.27 (95% CI 0.08-0.84) **p=0.02**	HR 0.26 (95% CI 0.09-0.79) **p=0.017**	HR 0.36 (95% CI 0.15-0.89) **p=0.027**
$\%\Delta ADC_{all}$ mean >18.0%	HR 0.62 (95% CI 0.31-1.23) p= 0.17	HR 0.35 (95% CI 0.12-1.02) p=0.05	HR 0.31 (95% CI 0.11-0.86) **p=0.024**	HR 0.35 (95% CI 0.15-0.83) **p=0.017**
$\%\Delta ADC_{b100}$ mean >18.1%	HR 0.53 (95% CI 0.27-1.08) p=0.08	HR 0.25 (95% CI 0.09-0.74) **p=0.01**	HR 0.21 (95% CI 0.07-0.60) p= **0.004**	HR 0.28 (95% CI 0.12-0.67) **p=0.004**
ΔADC_{all} at the 75th percentile >0.17x10^{-3} mm^2/s	HR 0.047 (95% CI 0.20-1.13) p=0.09	HR 0.46 (95% CI 0.12-1.84) p=0.27	HR 0.30 (95% CI 0.084-1.04) p=0.06	HR 0.25 (95% CI 0.09-0.72) **p=0.010**
ΔADC_{b100} at the 75th percentile >0.22x10^{-3} mm^2/s	HR 0.31 (95% CI 0.14-0.69) **p=0.004**	HR 0.37 (95% CI 0.11-1.27) p=0.11	HR 0.13 (95% CI 0.04-0.40) **p=0.0005**	HR 0.29 (95% CI 0.11-0.76) **p=0.012**
$\%\Delta ADC_{all}$ at the 75th percentile >13.4%	HR 0.54 (95% CI 0.24-1.19) p=0.12	HR 0.48 (95% CI 0.14-1.68) p=0.25	HR 0.28 (95% CI 0.08-0.91) **p=0.035**	HR 0.53 (95% CI 0.21-1.33) **p=018**
$\%\Delta ADC_{b100}$ at the 75th percentile >15.5%	HR 0.40 (95% CI 0.19-0.86) **p=0.017**	HR 0.26 (95% CI 0.08-0.82) **p=0.02**	HR 0.16 (95% CI 0.05-0.48) **p=0.0012**	HR 0.19 (95% CI 0.07-0.47) **p=0.0004**

Supplementary Material

Comparison of ADC calculation by vendor specific MRI

In this study 54.2% (26/48) of baseline MRI scans were performed on the Intera system, 39.6% (19/48) on the Aera system and 6.3% (3/48) on the Avanto system. 93.8% (45/48) patients had post NAC MRI performed on the same scanner i.e only 3 patients had post NAC MRI acquired on a different scanner during the follow-up period.

The ADC median values and pair-wise comparison by MRI system at baseline, post NAC, and ΔADC is given in supplementary material Table 6. The ADC_{all} mean and ADC_{b100} mean at baseline and post NAC were statistically significant between the Intera and Aera groups on Kruskal-Wallis Test, adjusted by Bonferroni correction for multiple testing. When ΔADC distribution was reviewed, no significant difference was seen between the scanners. Pearson Chi squared analysis demonstrated the distribution of NAC response status was not significantly different between the scanners and is unlikely to be a confounding factor.

Scanner switching for individual patients was avoided wherever possible. Individual patients therefore provide internal control of this variable when ΔADC is considered and that the changes seen with response are large enough to mitigate any scanner differences. However, when absolute ΔADC is summarised between different machines an error is likely to be introduced, as the degree of change between machines is different although not significant.

Supplementary Material

Table 6. Comparison of ADC between scanners used in the study

**MRI system-baseline (number of patients)**	**Baseline ADC**					
	**ADC_{all} mean x10^{-3} mm^2/s**			**ADC_{b100} mean x10^{-3} mm^2/s**		
	**median**	**range**		**median**	**range**	
Intera (26)	1.52	0.98	2.04	1.35	0.80	1.86
Aera (19)	1.21	0.85	1.68	1.09	0.78	1.50
Avanto (3)	1.04	1.01	1.43	0.97	0.79	1.24
	Pair-wise comparison (adjusted significance)* (p value)					
Intera-aera	0.033			0.023		
Intera-avanto	0.121			0.113		
Aera-avanto	1.00			1.00		
**MRI system- post NAC (number of patients)**	**Post NAC**					
	**ADC_{all} mean x10^{-3} mm^2/s**			**ADC_{b100} mean x10^{-3} mm^2/s**		
	**median**	**range**		**median**	**range**	
Intera (25)	1.52	0.98	2.04	1.35	0.80	1.86
Aera (22)	1.21	0.85	1.68	1.09	0.78	1.50
Avanto (1)	1.04	1.01	1.43	0.97	0.79	1.24
	Pair-wise comparison (adjusted significance)* (p value)					
Intera-aera	0.028			0.022		
Intera-avanto	1.00			1.00		
Aera-avanto	1.00			1.00		
**MRI baseline system (number of patients)**	**Absolute ADC change**					
	**ADC_{all} mean x10^{-3} mm^2/s**			**ADC_{b100} mean x10^{-3} mm^2/s**		
	**median**	**range**		**median**	**range**	
Intera (26)	0.36	-0.44	1.93	0.30	-0.21	1.41
Aera (19)	0.17	-0.02	1.04	0.16	-0.02	1.00
Avanto (3)	0.36	-0.12	0.84	0.47	-0.14	0.84
	Significance** (p value)					
Intera-aera	0.157			0.311		
Intera-avanto						
Aera-avanto						

* Kruskal-Wallis Test, adjusted by Bonferroni correction for multiple testing
** multiple comparisons not performed because no significant difference across samples i.e. distribution of absolute ADC change is same across all categories
